# Adaptive Immune Responses and Immunity to SARS-CoV-2

**DOI:** 10.3389/fimmu.2022.848582

**Published:** 2022-05-04

**Authors:** Dragan Primorac, Kristijan Vrdoljak, Petar Brlek, Eduard Pavelić, Vilim Molnar, Vid Matišić, Ivana Erceg Ivkošić, Marijo Parčina

**Affiliations:** ^1^ St. Catherine Specialty Hospital, Zagreb, Croatia; ^2^ Medical School, University of Split, Split, Croatia; ^3^ Faculty of Medicine, Josip Juraj Strossmayer University of Osijek, Osijek, Croatia; ^4^ Faculty of Dental Medicine and Health, Josip Juraj Strossmayer University of Osijek, Osijek, Croatia; ^5^ Medical School, University of Rijeka, Rijeka, Croatia; ^6^ Medical School REGIOMED, Coburg, Germany; ^7^ Eberly College of Science, The Pennsylvania State University, University Park, PA, United States; ^8^ The Henry C. Lee College of Criminal Justice and Forensic Sciences, University of New Haven, West Haven, CT, United States; ^9^ Institute of Medical Microbiology, Immunology and Parasitology (IMMIP), University Hospital Bonn, Bonn, Germany

**Keywords:** adaptive immunity, COVID-19, SARS-CoV-2, T cell, B cell, cellular response, humoral response, interferon gamma

## Abstract

Since the onset of the COVID-19 pandemic, the medical field has been forced to apply the basic knowledge of immunology with the most up-to-date SARS-CoV-2 findings and translate it to the population of the whole world in record time. Following the infection with the viral antigen, adaptive immune responses are activated mainly by viral particle encounters with the antigen-presenting cells or B cell receptors, which induce further biological interactions to defend the host against the virus. After the infection has been warded off, the immunological memory is developed. The SARS-CoV cellular immunity has been shown to persist even 17 years after the infection, despite the undetectable humoral component. Similar has been demonstrated for the SARS-CoV-2 T cell memory in a shorter period by assessing interferon-gamma levels when heparinized blood is stimulated with the virus-specific peptides. T cells also play an irreplaceable part in a humoral immune reaction as the backbone of a cellular immune response. They both provide the signals for B cell activation and the maturation, competence, and memory of the humoral response. B cell production of IgA was shown to be of significant influence in mediating mucosal immunity as the first part of the defense mechanism and in the development of nasal vaccines. Here, we interpret the recent SARS-CoV-2 available research, which encompasses the significance and the current understanding of adaptive immune activity, and compare it among naive, exposed, and vaccinated blood donors. Our recent data showed that those who recovered from COVID-19 and those who are vaccinated with EMA-approved vaccines had a long-lasting cellular immunity. Additionally, we analyze the humoral responses in immunocompromised patients and memory mediated by cellular immunity and the impact of clonality in the SARS-CoV-2 pandemic regarding breakthrough infections and variants of concern, both B.1.617.2 (Delta) and B.1.1.529 (Omicron) variants.

## Introduction

**Figure 1:**
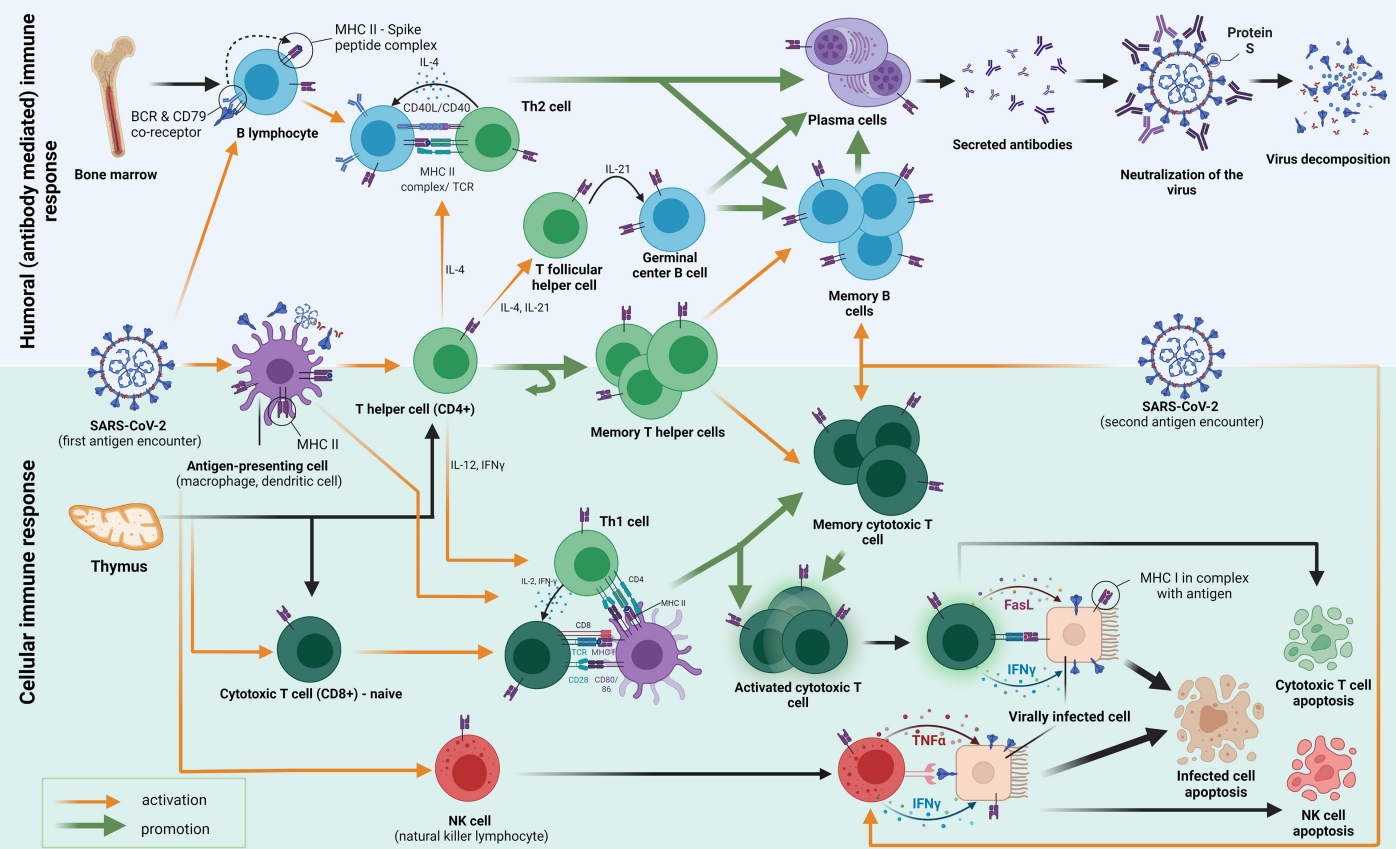
The figure shows an adaptive immune response to the first and second encounter with the SARS-CoV-2 antigen (spike protein). Following the first encounter, various antigens go through the process of phagocytosis and decomposition inside the antigen-presenting cells (APCs). APCs fragment the antigen into smaller peptides, which they present on their surface mediated by surface receptors called major histocompatibility complex class II (MHC-II) molecules. The antigens are then presented to several types of cells in the host, among which we emphasize CD4^+^ T helper cells (also B and CD8^+^ cells). B cells which differentiate into plasma cells, secrete antibodies that inhibit the entry of the viral particle into the healthy cells. The activation of T helper cells by APC causes them to differentiate into different subtypes with specific functions mediated by cytokine secretion and cell-to-cell contact. Th2-differentiated T helper cells help humoral responses mature by providing a second signal to B cells, mostly through IL-4 secretion and CD40/CD40L interaction. Some CD4^+^ cells also become T follicular helper cells (Tfh), which govern the important interactions in the germinal centers important for the maturation of memory B cells and long-lived high-affinity antibody-producing plasma cells. Another subset of CD4^+^ T cells differentiates into a pool of memory T helper cells. Th1 T helper cells play a crucial role in cellular response formation. They pivot the MHC-I activation of CD8^+^ cells (CTL, cytotoxic T lymphocytes) by interacting with APC’s simultaneously. Activated CTLs then act by causing apoptosis (by Fasl ligand–FasL binding) of the host cells that are infected with the SARS-CoV-2. Some CTLs differentiate into memory cytotoxic T cells, which have the role of fast restoration of the CTL response with secondary antigen contacts. A similar mechanism of destruction occurs when NK cells interact with the virally infected cell. They contain granules with IFNγ and TNFα in their cytoplasm, which, when secreted, cause programmed cell death. As well, the mechanism of activation of NK cells does not occur through MHC molecules is important because MHC is not always present on the virally infected cells.

At the beginning of an immune response to SARS-CoV-2, the exposure of human cells to the virus is the first step on the path (**Figure 1**). The initial encounter occurs in the upper respiratory tract through the nasal epithelium, tonsils, and adenoids—nasopharynx-associated lymphoid tissue (NALT) where mucosal immunity development is induced (1). Innate immunity acts first through pattern recognition receptors (PRRs) to pathogen-associated molecular patterns (PAMPs). Antiviral innate membrane (TLR7, TLR8, and TLR9) or cytosolic related receptors (RIG-I-like receptors) engage in strong antiviral type-I-interferon responses (2, 3). Additionally, primates evolved highly specialized cells for type-I-interferon production known as plasmacytoid dendritic cells (pDCs), which can be found in the blood and on the mucosa (4, 5). Besides the direct antiviral effects, type-I-interferon acts as the main link between the innate immune response and the activation of the adaptive immune response. Successful innate immunity activation results in limited viral entry, translation, replication, and assembly, helping identify and remove the infected cells, which provides all the requisites for the accelerated development of adaptive immunity (6). Once past the innate defenses, the virus is confronted by both major histocompatibility complex (MHC) classes I and II and direct natural killer (NK) cell activation. MHC-II molecules, embedded in the antigen-presenting-cell membranes (macrophages, monocytes, dendritic cells, and B cells), are important in activating both B cells, their proliferation and differentiation, and CD4^+^ T cells (7). Additionally, class II molecule expression in cells may be induced by interferon-gamma (IFNγ) and modulated by other factors, such as interleukin-4 (IL-4), interleukin-10 (IL-10), interferon-alpha/beta (IFN-α/β), tumor necrosis factor-alpha (TNFα), and glucocorticoids (8). Simultaneously, MHC-I is expressed on all cells containing a nucleus (including platelets) and serves mainly as an antigen recognition tool for CD8^+^ T cells (9). Natural killer cells represent 5–20% of circulating lymphocytes and 15% of total peripheral blood mononuclear cells in humans. A unique characteristic of natural killer cells is the ability to directly identify the infected cells, avoiding the necessity of the MHC-I complex presence (10–12). This attribute of NK cells puts them in an offensive position even against intracellular pathogens that evade CD8^+^ cells by interfering with MHC-I molecule expression (13). A bridge from the professional antigen-presenting cells to B cells, which produce antigen-specific antibodies, and CD8^+^ T cells is built out of T-helper cells (CD4^+^), mediated by the production of various cytokines, one of which is IFNγ (with IL-2, IL-4, interleukin-21 [IL-21], and TNF-α). The activation of special CD4^+^ naïve T cells by antigens induces migration from the T cell zone to germinal centers where they become T follicular helper cells (Tfh). Upon interaction with Tfh, follicular B cells undergo several processes such as isotype switching, somatic hypermutation, and rapid cellular division to seed germinal centers. Furthermore, Tfh interacts with other B cell subsets, playing a supporting role in the selection and survival of B cells. B cells differentiate into two types: long-lived, high-affinity antibody-producing plasma cells or germinal center-dependent memory B cells that react more rapidly with an antigen they have previously been immunized to (14). This also applies to SARS-CoV-2-specific Tfh cells that were expanded in patients with a mild or asymptomatic form of COVID-19. A higher frequency of a chemokine receptor CXCR3^+^ subset of T follicular help (TFH) cells was found in convalescent individuals who developed a severe form of COVID-19 (15, 16). A thorough examination of CD4^+^ and CD8^+^ T cells and B cells specific for various SARS-CoV-2 proteins indicated that coordinated activation of a humoral and cellular branch of the adaptive immune system mostly correlates with the milder COVID-19 form. Simultaneously, the uncoordinated response, observed in the elderly population, persists more frequently in patients that develop severe disease (17). Furthermore, another study found that a higher Th1/Th2 imbalance at hospital admission was associated with significantly increased mortality from COVID-19 in asthma patients, resembling steps toward cellular and humoral response development (18). A delayed humoral response and high IgG, IgM, and IgA levels reflect immunological imbalances that correlate with poor clinical outcomes (19–22). In non-survivors, a sudden decline of IgG antibodies against S1 and N proteins before death is observed (23). The collaboration between cellular and humoral immunity does not always have the same component layout. Depending on the cytopathic characteristics of the viral particle, some pathogens have a direct pathogenic effect, causing the death of the host cells in turn, thus demanding a strong and efficient antibody response that will limit the infection of host target cells. In contrast, the viral particle is a better substrate for cytotoxic T cell elimination when damage occurs due to the immune response-induced pathology of the host (24). Such divergence has not yet been established for SARS-CoV-2. Still, the latest research suggests that cellular immunity is of great value for a pool of patients who do not develop detectable SARS-CoV-2 IgG to the S1 protein after vaccination. This is demonstrated in patients receiving anti-CD20 monoclonal antibodies (Rituximab) immunosuppression therapy, which depletes B cells. However, few agammaglobulinemia patients develop pneumonia after SARS-CoV-2 infection (25–28). Relating to earlier strains in this millennium, the SARS-CoV infection also elicited memory B cells that tended to be short-lived compared to CD8^+^ T cell responses (measured six years after the infection); analog with memory T cells that have produced persistence even after 17 years of infection (29–31). In line with previous findings in a shorter period, investigation of antibody titers, both anti-S1 IgG and IgA responses, has shown a decrease over time. In contrast, anti-nucleocapsid antibody titers retained stability longer (from 40 to 159 days, median). Conversely, functional T cell responses remained robust, and it was even found that they had increased, in both abundance and potency, in the same timeframe (32).

Apparently, the memory mediated by CD4^+^ T cells developed in SARS-CoV-2 convalescent and/or vaccinated individuals is less prone to being avoided by mutations occurring in some variants of concern (VOC), while humoral immunity (neutralizing antibody function) may be partially avoided (33–37). There is also an indication that the key to understanding protection against these VOCs may be hidden in cross-reactive SARS-CoV-2-specific T cell-mediated immunity (33).

Observation of the general population reveals the persistence of many cases of seronegative, unexposed, and asymptomatic individuals who are protected by developed SARS-CoV-2-specific cellular immunity, which can be detected with proper laboratory tests like interferon-γ release assay (IGRA) to spike protein (ELISpot) (38). Playing a role in the innate immune response, IFNγ is produced both by natural killer cells (NK) and natural killer T cells (NKT), and has an important task in the CD4^+^ and CD8^+^ cytotoxic T lymphocyte (CTL) effector T cell function (“type 1” immune response) once an antigen has been specified and adaptive immunity develops (39). To define IFNγ secreted by Th1 CD4^+^ cells with IL-12, it is considered the main CD8^+^ T cell differentiation cytokine; simultaneously, the dominant B cell-activating cytokine is IL-4, secreted by Th2 CD4^+^ cells (40, 41).

Since a thoughtful response is of a significant influence and is crucial for further understanding the SARS-CoV-2 pandemic, the role of determining the cellular immune response may provide data that, especially concerning humoral immunity, could add value to our knowledge. Higher understanding could provide us with better vaccines and vaccination protocols. This also includes social measures—resolving asymptomatic and immune individuals from unnecessary isolation and testing, as well as preventing the spread—and possibly predicting clinical outcomes.

In this outline, emphasis is put on the importance of T-cell response detection compared to B cells and humoral immunity measurements. Following, the T-cell response is assessed in more detail, with its role in immunological memory and the pathophysiology of COVID-19. To gain further depth in understanding of cellular immunity, IFNγ production and measurement are assessed together with its interactions as a back-and-forth communication molecule between adaptive and innate immune responses. Lastly, the cross-reactivity of T cells and antibodies is evaluated between previous coronaviruses and SARS-CoV-2 with an eye towards the emerging variants of concern. Mucosal immunity has been appraised in the context of subject matter, however, in a less thorough manner. Also, the immune responses occurring in pregnancy have not been analyzed.

## T Cell Immunity Existence Despite Impaired Humoral Response Detection

Since memory B cells and serum immunoglobulins are associated with disease severity and mortality in patients with COVID-19, it is essential to examine what happens when this humoral part of the immune response is impaired. To be more precise, more severe disease and deceased patients have significantly lower levels of total B cells, naive B cells, switched memory B cells, and serum IgA, IgG, IgG1, and IgG2 than recovered patients (42). Furthermore, the persistence of neutralizing antibodies was associated with greater disease severity (43). At first, neutralizing antibody titers were shown to decline during the early recovery of most of the asymptomatic and symptomatic groups previously infected, suggesting at first that the effectiveness of neutralizing the humoral response induced by vaccines may also be relatively limited (44). Nonetheless, vaccinated participants have appeared with significantly higher neutralizing antibody titers than patients with previous COVID-19 (median was 169 and 139 days after diagnosis was set in mild and moderate-severe presentations) (45). Other epidemiological data also demonstrated that both two doses of the Pfizer–BioNTech (BNT162b2) (90%) and AstraZeneca (Vaxazevria) (79%) COVID-19 vaccine did preserve their highly protective attributes against hospitalization and death even after six months of the second dose (46). Moreover, neutralizing antibodies are also emphasized in the SARS-CoV-2 B.1.617.2 (Delta) variant infections. Information has been gathered and several papers report the existence of 25 and 100 times higher neutralizing antibody responses against B.1.351 in individuals who were vaccinated after COVID-19 infection, although neither the infection nor the vaccine contained the B.1.351 spike protein, in contrast to vaccination alone and infection alone, respectively (47). It may be worth pondering deeper than analyzing antibody neutralization levels solely since they wane quickly but establish high levels in different scenarios as described earlier.

Since measuring only humoral responses does not offer satisfying answers; the scientific community has started to include cellular immunity evaluation in contemporary studies. For example, a comparative immunogenicity investigation of a homologous or a heterologous third dose of seven vaccines was observed to incorporate both immune branches and validate the necessity of complementary B and T-cell activity, which we had described in the introduction (48).

Considering the most endangered COVID-19 population, a Huzly et al. study involved 149 immunocompromised individuals in gathering important information about their T-cell response to vaccination (38). The results varied among different types of immunosuppression, and unfortunately, most of them displayed an inadequate response to vaccination. However, some of them did mount a T-cell response despite an undetectable humoral component, which was the most obvious (13 of 20 patients) in drug-depleted B-cell patients. Another study compared patients with hematological cancer (impaired humoral immunity) who also had COVID-19 and noted improved survival in those with more CD8^+^ T cells, including those treated with anti-CD20 therapy (49). In contrast, those who received organ transplantation hadn’t produced such feedback, corroborating the work of Sattler et al., which logged no alloreactivity promoted by BNT162b2 in kidney transplant patients (50). Thankfully, recent research found that after the third dose of the SARS-CoV-2 mRNA-1273 vaccine, 49% of kidney transplant patients who were non-responders to 2 doses of the vaccine, eventually developed seropositive status (51). To elaborate further, an appraisal of immune responses after the second mRNA SARS-CoV-2 vaccine dose in a category of oncologic patients with multiple myeloma was done, comparing CD4^+^, CD8^+^, and anti-S IgG antibody concentrations. Both CD4^+^ (35%) and CD8^+^ (28%) responses were noted as similarly occurring among those with seronegative response status to the vaccine suffering from this plasma cell malignancy. They have also shown a correlation that demonstrates similar levels of CD4^+^ and CD8^+^ immunity in seropositive patients compared to healthy vaccinated donors. Since this is one of the most sensitive groups to infectious disease, the same study results indicate the importance of measuring both response types in different therapy regimens, showing variability between them and choosing the best-personalized approach for each individual (52). A recently published valuable example of a T-cell response epitope peptide-based vaccine entered the phase II clinical trial conducted in the patient population with B-cell/antibody deficiency (53). However, in a study concerning the Indians, immune memory was detected among mild COVID-19 patients (28 participants) up to 5 months after recovery in both CD4^+^ T and B cells, with a minimal contribution from CD8^+^ T cells (54).

In the summer of 2020, a small cohort of patients exhibited even 40% asymptomatic individuals and 12.9% of those recovering from mild COVID-19 no longer had antibodies 56 days after the hospital release date (44). Several papers mention a manifestation of seronegative patients having confirmed T-cell response in slightly different scenarios. One of those came across 17% of patients who were positive on the IFNγ-ELISpot assay and had a previously confirmed PCR of SARS-CoV-2 with borderline or negative IgG testing levels 60 days after symptom onset (25). Another French study analyzed intrafamilial exposure and gathered data (between 49 and 102 days after symptom onset) from 11 seronegative and RT-PCR-negative household contacts. Four developed T-cell responses, suggesting that virus-specific T-cell reactions without seroconversion have occurred (55).

However, antibody decline and their impaired measurement do not necessarily imply that there is no humoral immunity. The RBD and N protein-specific B memory (Bmem) cells continued to increase until 150 days post-symptom onset, despite a decline in serum antibodies. A significant correlation to T-cell immunity implies the fact that RBD-specific Bmem numbers are significantly correlated with circulating follicular helper T cell numbers. The analysis of SARS-CoV-2-specific Bmem cells seems to enable the detection of long-term immune memory following infection or vaccination for COVID-19 (56, 57). As well, research suggests that anti-S antibody titers correlate with the frequency of spike protein-specific bone marrow plasma cells, which both show a rapid (within the first four months) and then a gradual decline (up to 11 months) after the infection. This suggests memory properties of these plasma cells (58). Furthermore, breakthrough infections lead to an increase in specific antibodies in serum and saliva associated with memory B cells (59).

Measurements of the humoral response to COVID-19 have been carried out extensively. Less examined is mucosal immunity, which seems to exert its protective role mainly through antibody neutralization of viral particles. In the mucosal subepithelium and associated glands, mucosal plasma cells produce IgA, which is transported into the secretions, and released as SIgA (secretory IgA). Except for neutralization, SIgA may also inhibit viral adherence to and invasion of epithelial cells, cause agglutination, and facilitate the removal of mucus. Additionally, some B cells that are induced in the tonsils travel to peripheral lymphoid tissue to secrete circulatory IgG (60). Specific IgA serum concentrations decrease within one month of the onset of natural infection symptoms, while neutralizing IgA remain detectable in saliva for a longer time (2–3 months) (61).

The vaccine development efforts were mainly focused on circulatory IgG and CTL while having an impaired mucosal IgA response. A peculiar interest has been attributed to the development of mucosal immunity-inducing vaccines. Intranasal vaccination boosts with adenoviral vectored vaccines resulted in the systemic and mucosal immunity of mice, leading to complete SARS-CoV-2 protection. Compared with the double intramuscular application of the mRNA vaccine, intranasal boosts induced high levels of mucosal IgA and lung-resident memory T cells, with mucosal neutralization of VOC also being enhanced (62). After the single dose, another adenoviral vectored vaccine completely protected mice from the lethal SARS-CoV-2 challenge, preventing weight loss and mortality. It induced the production of mucosal IgA in the respiratory tract, serum neutralizing antibodies, and CD4^+^ and CD8^+^ T cells with a Th1-like cytokine expression profile. The immunity was sustained for more than six months (63).

## Role of T Cells in COVID-19 Immunological Memory and Viral Pathophysiology

We may speculate that the interest in researching the role of cellular immunity memory in the COVID-19 pandemic first arose from the ancestors of the disease. This knowledge lends to the notion that a general feature of coronavirus infection is transient humoral immunity, with vaccination-induced humoral immunity data from the 2019 pandemic presenting that two-dose antibody response may wane in Israeli adults aged ≥60, with the third dose being beneficial (29, 64–66). Furthermore, recent research among the Croatian population shows that the key to long-term protection is cellular immunity. In comparison with the study participants who were previously infected with SARS-CoV-2 (group 1) and those vaccinated with EMA-approved vaccines (group 2), it was proved that the ones who had both been vaccinated and recovered from COVID-19 (group 3) had significantly higher levels of cellular immunity and antibody titers. The same research found that previously infected and vaccinated individuals had long-lasting cellular immunity. Furthermore, antibody levels negatively correlated with time since the last contact with a viral antigen, while cellular immunity showed a persistent relationship even within 20 months (67). After the infection has been successfully warded off, a part of the defensive strategy is saved long-term for future use in the form of memory T cells. This was confirmed by an investigation of SARS-CoV-2-specific memory T-cell responses that were maintained in COVID-19 convalescent patients for up to 10 months post-symptom onset regardless of disease severity. Also, stem cell-like memory T cells were increased (68). They remain ready to mediate an augmented response to the pathogen by eliciting new effector cells. Some of those differentiate into cellular immunity mediators, which then mount an attack on the infected, but also, as previously mentioned, T follicular helper (Tfh) cells are crucial for enhanced B-cell response in the case of pathogens evolving and trying to elude antibody recognition (69).

Sekine et al. observed that plenty of individuals, not necessarily seropositive, with an asymptomatic or mild disease course of COVID-19 had excellent memory T-cell responses (70). This information is based on their discovery of a SARS-CoV-2-specific T-cell early differentiated memory phenotype (CCR7^+^ CD127^+^ CD45RA^−/+^ TCF1^+^) associated with stem-like properties, which had previously been seen in the history of other viral pathogens and successful vaccines (71–75). Additionally, Huzly et al. state that memory mediated by CD4^+^ T cells developed in SARS-CoV-2 convalescent and/or vaccinated individuals is less prone to being avoided by mutations occurring in some variants of concern (38).

As we know, some viral pathophysiology is linked to the magnitude of inappropriate immune response to a greater extent than direct cytopathic effect (analog to chronic HBV infection). With severe COVID-19 being associated with inflammation of the lungs, an important question is whether T cells always play a protective role in the SARS-CoV-2 infection, or whether they may possess unwanted properties that significantly contribute to disease pathology. Another interesting problem in severe COVID-19 patients stems from the fact that those individuals have marked lymphopenia, which, with the study that specifies a reduction of CD8^+^ T cells in the bloodstream with worse prognosis, suggests that the primary T cell activity is not located in the bloodstream but in the lungs instead (76–78). Hence, a segment of new research is directed more toward memory T cells (longevity, biomarkers, and other characteristics) specific to the lung tissue rather than systemic circulation only (79, 80). In convalescent patients, lung-tissue resident memory T cells are frequently detected even ten months after initial infection, in which time-correlating blood does not reflect tissue-resident profiles (81).

Furthermore, a multi-target CD8 T-cell peptide COVID-19 vaccine targeting several structural (S, M, and N) and non-structural (NSPs) SARS-CoV-2 proteins (with selected epitopes from conserved regions) was examined. The vaccination induced a substantial proportion of virus-specific CD8 T cells expressing the specific phenotype of tissue-resident memory T lymphocytes (CD103, CD44, CXCR3, and CD49a) (82). Bertoletti et al. showed that the existence and magnitude of severe COVID-19 cases is related to the inflammatory events (the activation and recruitment of myeloid cells into the airway, production of inflammatory cytokines, and complement activation), which seem to connect directly to the viral load or an immune response with uncoordinated features (83–85). However, evidence suggests that these events may be linked to parts of immunity rather than only SARS-CoV-2-specific T cell activation, such as complement activation (86). Additionally, IgG dominance has been associated with severe COVID-19.

In contrast, mucosal SIgA is essentially non-inflammatory, even anti-inflammatory, in its mode of action. IgA does not activate complement by the classical pathway-specific for IgG. Moreover, the IgA antibodies have even been shown to inhibit complement activation mediated by IgM or IgG antibodies (60). Hence, a stronger mucosal immunity could theoretically avoid some severe COVID-19 pathology. Bertoletti et al. also compared the two studies and noted that significant virus-specific T-cell responses in symptomatic and asymptomatic patients are independent of the presence of symptoms (70, 87). However, SARS-CoV-2-specific T cells in asymptomatic patients were functionally superior to those detected in patients with symptoms, presuming that effective T cell response may be a part of an explanation and possible prediction of the severity of the disease (88).

The vaccine research is interesting because upon SARS-CoV-2 spike peptide interaction with the T-cell receptors (TCRs) of lymphocytes, CD4^+^ cell-fraction can be purified and inserted into allogeneic CD4^+^ T cells, which can activate them and induce IFNγ *in vitro*. Also, it was stated that CD4^+^ T cells appeared at a higher frequency and with more numerous corresponding epitope presentations than CD8^+^ T cells in the recovered COVID-19 patients (89).

## An Essential Role of IFN-γ in Facilitating and Interpreting SARS-CoV-2-Specific Cellular Response

### IFN-γ Facilitation of SARS-CoV-2 Specific Cellular Response

IFN-γ, representing the only member of the type II class of interferons, is a protein produced most abundantly by NK cells, type 1 CD4^+^, CD8^+^, and gamma delta T cells, and to a lesser extent by NKTs, B cells, and professional APCs (41). One of the primary roles of IFN-γ in cellular immunity is pathogen killing. IFN-γ, on the other hand, activates M1 macrophages and induces macrophage production, namely, various inflammatory mediators, reactive oxygen, and nitrogen intermediates, and increases macrophage expression of MHC antigens, which facilitates antigen presentation to T cells. However, IFN-γ receptor expression in T cells determines their response rate. IFN-γR1 (α) and IFN-γR2 (β) are the two chains that make up the IFN-γ cell-surface receptor. The α (R1) chain is sufficient for binding, but the β (R2) chain is required for signaling and receptor complex formation. Both Th1 and Th2 cells express IFN-γR1, but only Th2 cells express IFN-γR2.Therefore, Th1 cells produce but do not respond to IFN-γ, whereas Th2 cells do not make this cytokine nor respond to it. Macrophages express both receptor chains and are an important target of IFN-γ activity (90).

Discussing the pathophysiology occurring in severe COVID-19 cases, while interferon may be protective during the early stages of the disease, persistent IFNγ production in the presence of an inflammatory lung macrophage signature could ultimately drive macrophage hyperactivation (91). Interactions like this are essential due to the tendency to oversimplify the distinction between the adaptive and innate components of the immune system. Instead, we shall think of examples implying adaptive characteristics of innate response cells—epigenetic changes that occur during macrophage activation underlie the long-term imprinting of macrophage responses to microbial encounters, namely, SARS-CoV-2, and the one representing IFNγ being the back-and-forth communication molecule in the case of SARS-CoV-2 infection among adaptive and innate immune responses (92, 93). Epigenetic changes have also been described as part of the SARS-CoV-2 immune evasion mechanism regarding innate and adaptive immunity. Specifically, the viral 2′-O-Mtases mimic the cap1 structure of the host and cause immune evasion through a Hsp90-mediated epigenetic process to hijack the infected cells *via* autophagy (93). Additionally, unlike antigen-specific T-cells receptor biological properties, natural killer cells do not use clonotypic receptors; however, a relatively small population of memory NK cells has been described as long-lived effectors capable of rapid recall responses to secondary pathogen encounters (94).

Furthermore, observations have been made that NK cells contribute to antiviral immune mechanisms and secrete IFNγ (95, 96). Subsequently, NK cells possessing antiviral effector properties have been characterized by the NKG2C receptor (among others), potentially setting boundaries to the degree of SARS-CoV-2 infections. Additionally, a recent genetic study reveals that genetic variants in the NKG2C/HLA-E axis have a significant impact on the development of severe SARS-CoV-2 conditions (97). NKG2C is an activating NK cell receptor that leads to NK cell activation following binding to HLA-E on infected cells. They have found that the deletion of KLRC2, the gene encoding the NKG2C receptor, and to a lesser degree, the HLA-E*0101 allele, encoding HLA-E, were significantly overrepresented in hospitalized patients (particularly ICU patients), compared to those with the milder form of SARS-CoV-2. Both genetic variants were independent risk factors for severe COVID-19. More research is needed to determine whether these deletions have a significant impact on cellular immune response, IFNγ secretion measurements, and result interpretation.

### IFN-γ in the Interpretation of SARS-CoV-2-Specific Cellular Response

The SARS-CoV-2-specific interferon-gamma release assay (IGRA) is based on the measurement of IFN-gamma levels in the collected blood samples after stimulation with specific peptides. Here, the peptides are made from the S1 domain of the SARS-CoV-2 spike protein (which mediates the entry of the SARS-CoV-2 virus into the host cell) (98, 99). In the event of a present and competent cellular immune response in the whole heparinized blood of the patient, the T cell receptor is directly elicited with the peptides, which causes IFNγ production and secretion. Subsequently, quantitative IFN-gamma detection is performed on the plasma portion of the whole heparinized blood (38).

A commercially available variant of this test (EUROIMMUN), when measured and combined with a particular, well-validated cut-off strategy, demonstrated a specificity of 96.3–100% and a sensitivity of 75.4–89.6%. The difference in the sensitivity values depends on whether the test was done less than or more than 6 months after the infection or vaccination and on the different cut-off values. Using this assay, an appraisal of functional cellular immunity response from five distinct cohorts was made and the response frequencies decayed from severe COVID-19 convalescents (100% of individuals), followed by mild COVID-19 convalescents (87%), exposed family members (67%), and healthy donors (46%) (70). Reynolds et al. provided us with valuable proof of what is being mentioned in scientific surroundings with an increasing frequency known as “hybrid immunity,” also called, in dramatic fashion, “super-immunity” (100, 101). They demonstrated that individuals vaccinated with one dose after prior infection with SARS-CoV-2 had superior T-cell immunity, antibody-secreting B-cell response, and antibodies that neutralize B.1.1.7 and B.1.351 compared with naive vaccinated individuals. However, the term was previously created by sources focused on measuring humoral responses to SARS-CoV-2, and now we know that it affects the cellular branch as well (102). IFNγ concentrations after the first dose of the BNT162b2 vaccine were similar to those after a recent infection (<6 months) and significantly lower than those after the second dose (38). In this regard, we may hypothesize that IFNγ measurement combined with a humoral response unit count could provide sufficient information to the posed question of the Centers for Disease Control and Prevention at the time of writing. Present data is insufficient to determine an antibody titer threshold that would indicate when an individual should be protected from SARS-CoV-2 infection and need not undergo additional vaccination. For the time being, their current recommendations for everyone aged 18 or older find Pfizer-BioNTech and Moderna boosters eligible six months after the second dose and Johnson & Johnson’s Janssen two months after the first dose. Retrospectively, in the past two years, the humoral response has been thoroughly examined, from which it was found that antibody levels cannot give us explicit assumptions about the development of spike-specific T cells, especially in the late convalescent phase (8–9 months) (43, 103, 104).

Analysis from the first year of the pandemic stated that neutralization ability correlated positively with anti-S IgG or anti-RBD IgG (105). The FDA’s notice and recent JAMA medical reports observe that, out of all antibodies having binding ability, only some neutralize, and very few authorized clinical tests can distinguish between the two classes (106). Results from 2 studies report that viral-specific humoral responses to SARS-CoV-2 (IgG) decreased significantly within the first six-month period but maintained stability for up to 1 year following hospital discharge (107, 108). It was also demonstrated by Feng et al. that SARS-CoV-2-specific IFNγ secreting cells (spike and nucleoprotein-specific) decreased at 6-month follow-up and stayed stable during 12-month visits. Additionally, the distinction between the cellular response to SARS-CoV-2 in participants who have been vaccinated and/or infected and uninfected healthy donors could be obtained with a significant degree of sensitivity and specificity by measuring plasma TH1-type IFNγ^+^ and IL-2^+^ effector cytokines from SARS-CoV-2 peptide-stimulated whole blood to SARS-CoV-2 (109).

## Cross-Reactivity of SARS-CoV-2-Specific T Cells

In the recent coronavirus pandemic (COVID-19), there are still several questions and unpredictable manifestations of the kinetics and severity of the disease, ranging from asymptomatic to patients subjugated to the pathogen. Nevertheless, scientists are emerging with new data that will help understand the contribution of cross-reactivity in the T lymphocyte population. To begin, they estimated that 34% of healthy donors have SARS-CoV-2-specific S-cross-reactive CD4^+^ T cells (peripheral blood mononuclear cell (PBMC) and plasma samples collected 2015–2018), compared to 40–60% of unexposed individuals (110, 111). Following this, Mateus et al. discovered that circulating “common cold” coronaviruses (namely, OC43 and 229E) have particularly homologous sequences to SARS-CoV-2-specific CD4^+^ T cells. Similar results were found in another study concerning the Indian population, specifying that 66% of unexposed study participants (42 total participants) had SARS-CoV-2 cross-reactive CD4^+^ T cells (54).

Additionally, it has been investigated that the ELISpot IFNγ assay in unexposed individuals had a T-cell response more specific to non-structural proteins from the ORF-1 region (NSP7 and NSP13 dominantly), while convalescent donors had their T-cell response tailored for structural SARS-CoV-2 proteins (31, 112). A recent study emphasized that about 80% of the epitopes have not been previously seen in unexposed compared to exposed donors, suggesting that overlap among them exists. Still, the substantial difference is persistent in the SARS-CoV-2-cross-reactive memory T-cell repertoire (113). The S protein sequencing (the most antigenic part of the virus) of nine strains of the coronavirus obtained that the region from amino acid residue numbers 945 to 1,100 is highly conserved. This is important for vaccine development considering that several potential epitopes recognized by B and T cells were identified in that region (114).

However, the pattern of CD4^+^ cross-reactivity to different epitopes was inconsistent enough to enable specifying them and drawing clear conclusions. Yet, we are capable of distinguishing different patterns of immunodominance. CD4^+^ T cells were shown to be more responsive to M, S, and N antigens co-dominantly (11–27% each), while CD8^+^ cells also targeted S protein, but it was not a particularly dominant response. CD8^+^ cells demonstrated a response just as strongly to other viral proteins—nsp6, ORF3a, and N—which accounted for nearly 50% of the total CD8^+^ T cell response (111). The matter seems more complex in that the T-cell response recognizes at least 30–40 epitopes in each donor (1,925 different overlapping peptides spanning the entire viral proteome were used) (113). Another study compared other coronavirus T-cell-specific immunity. It established that S-protein is considered responsible for two-thirds of CD4^+^ responses while N and M have limited or no reactivity at all (115).

From previous research, we can observe that a significant degree of T-cell memory heterogeneity exists in correspondence to the past exposures of the immune system of the patient. Whether this immune modification and how it influences the course of COVID-19 and the vaccination trajectory is unknown, more research will be required. A significant benefit of determining cellular response specificity is also winning the race against emerging notorious strains such as B.1.1.7, B.1.351, B.1.617.1 and B.1.617.2, B.1.525, B.1.1.28.1, B.1.1.529 (Delta strain) and most recently added to the VOC list by WHO—B.1.1.529 (Omicron strain) (116–120). Kojima and Klausner reviewed published biological studies (including the recent period of predominantly delta variant spread) and found significant results, which documented that previous infection provides remarkable immunity with a shallow reinfection incidence rate and hospitalization frequency (121). An Israeli population epidemiological analysis has concluded that SARS-CoV-2-naïve vaccinated people had a 13.06 times increased risk of breakthrough infection (an infection that occurs in fully vaccinated people) with the B.1.617.2 variant compared to those who were COVID-19 convalescents (122). Moreover, Primorac et al. found that out of 200 study participants, only 1 individual who recovered from COVID-19 (0.5%) was re-infected, while 6 participants (3%) were affected by breakthrough SARS-CoV-2 infection after receiving the vaccine (67). While investigating the responses of children to coronavirus infection, a study concluded that humoral and cellular responses remain stable even after six months compared to humoral waning in adults. The S2 domain is more highly preserved among human coronaviruses than S1. This is compatible with hCoV-specific antibodies in children that target more conserved epitopes, and have the potential for neutralizing activity against SARS-CoV-2 (123). Nevertheless, more effort is required for long-term characterization of the T cell response in children, which has great potential since they usually present with mild or asymptomatic COVID-19 (124).

In the United States, an epidemiological study demonstrated that effectiveness against symptomatic infection ≥7 days after two doses was 89–92% against Alpha, 87% against Beta, 88% against Gamma, 82–89% against Beta/Gamma, and 87–95% against B.1.617.2 across vaccine products (BNT162b2, mRNA-1273, and ChAdOx1) (125). The assumption of whether and how the vaccine may resemble long-term protection against VOC has not been examined thoroughly yet. It is worth considering that the analyzed data proves effective vaccine protection against hospitalization (almost 90%) even after the B.1.617.2 variant spreads among the population (46, 126, 127). Another more extensive study (960,765 household contacts) demonstrated that the likelihood of household transmission was approximately 40 to 50%, resembling vaccine protection against infection (128). Be that as it may, cellular immunity recognizes various viral epitopes, not only the S-protein. Most mutations occur; it could be a crucial defense mechanism against an evolving threat. Therefore, vaccination goals should be aimed at inducing durable and effective responses mediated by our adaptive immune system’s humoral and cellular components (107). Subsequently, a novel T-cell response epitope-targeted vaccine had a more prominent cellular response than the previously approved vaccines until month 3, opening doors for further creativity in vaccine development (53).

This is boosted by the specimens first collected on 8 November 2021, in South Africa, imposing the emergence of the Omicron variant (B.1.1.529), which demonstrated 44 amino acid substitutions, six amino acid substitutions, and one amino acid insertion after sequencing was done. Most of these alterations had been associated with localizations at 33 of the 1,273 amino acid residues of the S-protein (15 found in the receptor-binding domain, which is critical for viral binding and represents the main target of most vaccines). They have affected regions that may enhance viral spread, decrease neutralizing antibody binding ability, and increase viral RNA expression. Some have shown reduced susceptibility to available monoclonal antibody therapeutics in previous viral variants (129–131). A recent report stated that identifying the Omicron variant is possible by using RT-qPCR assays that target Omicron characteristic mutations in the nsp6 (Orf1a), spike, and nucleocapsid genes (132). Since the other coronavirus NSP6 protein has previously been accredited with the ability to limit autophagosome expansion, the mutation occurring in this area could play an essential role in enhancing the SARS-CoV-2 Omicron variant defense mechanisms against the immune system of the host (133).

Nevertheless, recent studies show that the Omicron variant is causing fewer hospitalizations and a decrease in severity and mortality (134). Research conducted in other VOCs demonstrated variant cross-neutralization following B.1.617.2 (Delta) strain breakthrough infection compared to non-Delta VOC (B.1.1.7, B.1.351, P.1) breakthrough infection, the original SARS-CoV-2 strain (WA1), and 2-dose vaccinated individuals (BNT162b2). This implies that booster dose vaccine effectiveness may be enhanced by inserting VOC-specific peptides into vaccines (135).

## Discussion

Considering our limited knowledge due to the momentum at which the COVID-19 pandemic developed, along with the enormous heterogeneity of the response to the viral particle and other factors (such as “pulmonary escape” generating generalized lymphopenia), we must emphasize that more research will need to be carried out to better understand cellular immunity in the case of SARS-CoV-2. However, determining the functional cellular response by using the IFNγ ELISpot assay to spike antigen was found to be an effective tool in determining the T-cell response status of an individual (67). As far as we are aware, an approach that encompasses the T-cell response and an assessment of virus-specific antibodies provides a powerful tool to measure COVID-19 immunity to a higher degree of confidence than either quantification on its own (109). This finding may allow us to develop a more sophisticated vaccine that would elicit more durable, mutation-resistant (targeting epitopes with a lower frequency of mutating) and specific responses and a personalized vaccination protocol. This is particularly important since the emergence of a novel B.1.1.529 (Omicron) variant whose genome has been shown to be altered in a region previously primarily targeted by vaccine manufacturers. However, it is encouraging that most vaccine-induced SARS-CoV-2 T cells were reactive against conserved regions of mutant S-protein, which could provide protection even against the VOC. A profound decision-making process should spare you from unnecessary vaccination and its side effects. The approved SARS-CoV-2 vaccines show a good safety profile; however, caution should not be neglected. Mice vaccinated with four different SARS-CoV vaccines were challenged with the SARS virus and all animals developed Th2-type immunopathology, suggesting hypersensitivity to SARS-CoV components (136). Still, more importantly, better decision-making would determine when one needs to be revaccinated, for example after the humoral response has fallen below detection levels or help prove low-positive/gray zone antibody results. The immunocompromised are one of the vulnerable groups that could benefit from a better understanding of the SARS-CoV-2-specific T cell immunity and its vaccine. Being optimistic, we mentioned an example of a T-cell response-epitope aimed peptide vaccine, which demonstrated a good safety profile in a phase I open-label trial and induced a VOC-independent, more extensive, and more robust cellular immune response compared to the previously SARS-CoV-2 infected or vaccinated with approved vaccines, transiting towards a phase II trial for patients with B cell/antibody deficiency (53). Nevertheless, patients having deficient immunity are the ones who can provide us with some of the most valuable discoveries since they represent immunological extremes from which we can draw assumptions more easily.

Mucosal immunity has also been established to play a pivotal role in the immune response. It may lower the risk of transmission by decreasing the level or duration of viral shedding from infected individuals (137). This could be very beneficial with newly emerging VOCs that seem to acquire an increase in transmission potency (B.1.617.2 vs. B.1.1.529). Since mucosal immunity is partly compartmentalized, vaccines should include immunization through NALT in the upper respiratory tract to produce a strong response that could prevent infection at the site where the first contact occurs (60). Several vaccines that offer complete protection from infection or lethal challenges in mice have already been developed (62, 63). Lastly, in response to novel breakthrough infections occurring in vaccinated individuals, the necessity of protection from them, and the public health challenges (booster doses particularly), Lipsitch et al. examined the literature (138). They stated that the rates of breakthrough infection are best seen as a consequence of the level of immunity of an individual at any given moment concerning the variant and disease severity which further emphasizes the importance of complete knowledge of the immune level of a person including both branches, cellular and humoral.

## Author Contributions

Conceptualization, DP and MP. Writing—original draft preparation, KV, EP, and PB. Writing—review and editing, DP, IEI, VilM, VidM, MP, PB, and EP. Visualization, VilM, PB, and KV. Supervision, DP and VidM. Project administration, DP. All authors listed have made a substantial, direct, and intellectual contribution to the work and approved it for publication.

## Conflict of Interest

The authors declare that the research was conducted in the absence of any commercial or financial relationships that could be construed as a potential conflict of interest.

## Publisher’s Note

All claims expressed in this article are solely those of the authors and do not necessarily represent those of their affiliated organizations, or those of the publisher, the editors and the reviewers. Any product that may be evaluated in this article, or claim that may be made by its manufacturer, is not guaranteed or endorsed by the publisher.
